# Fn14 deficiency ameliorates psoriasis-like skin disease in a murine model

**DOI:** 10.1038/s41419-018-0820-6

**Published:** 2018-07-23

**Authors:** L. Peng, Q. Li, H. Wang, J. Wu, C. Li, Y. Liu, J. Liu, L. Xia, Y. Xia

**Affiliations:** 1 Department of Dermatology, The Second Affiliated Hospital, School of Medicine, Xi’an Jiaotong University, Xi’an, China; 2 Core Research Laboratory, The Second Affiliated Hospital, School of Medicine, Xi’an Jiaotong University, Xi’an, China

## Abstract

Tumor necrosis factor (TNF)-like weak inducer of apoptosis (TWEAK) is a multifunctional cytokine that acts through its receptor fibroblast growth factor-inducible 14 (Fn14). Recent studies demonstrated that the TWEAK/Fn14 signals participate in the development of psoriasis. The purpose of this study was to further explore the effect of Fn14 inhibition on experimental psoriasis. Psoriasis-like skin disease was induced in the wild-type and Fn14-knockout BALB/c mice. We found that Fn14 deficiency ameliorates psoriasis-like lesion in this model, accompanied by less inflammatory cell infiltration and proinflammatory cytokine production in lesional skin. The cutaneous expression of TNF receptor type 2 also decreased in the Fn14-deficient mice. Moreover, the topical application of TWEAK exacerbated psoriatic lesion in the wild-type but not in the Fn14-deficient mice. Furthermore, TWEAK promoted the expression of interleukin 8, keratin 17, and epidermal growth factor receptor (EGFR) but inhibited the expression of involucrin in psoriatic keratinocytes in vitro. Interestingly, such effect of TWEAK was abrogated by an EGFR inhibitor (erlotinib). TWEAK also enhances the proliferation and interleukin-6 production of dermal microvascular endothelial cells under psoriatic condition. In conclusion, TWEAK/Fn14 signals contribute to the development of psoriasis, and involves the modulation of resident cells and the transduction of the EGFR pathway. Fn14 inhibition might be a novel therapeutic strategy for patients with psoriasis.

## Introduction

Tumor necrosis factor (TNF)-related weak inducer of apoptosis (TWEAK) is a member of the TNF family. TWEAK acts through binding to its sole receptor fibroblast growth factor-inducible 14 (Fn14). Fn14 is widely expressed in various types of resident cells including dermal microvascular endothelial cell, dermal fibroblasts, and epidermal keratinocytes^1–5^. Under physiological condition, Fn14 is weakly expressed in resident cells or tissues. However, inflammatory responses or malignant microenvironments can upregulate expression of both TWEAK and Fn14^6,7^. The interplay between TWEAK and Fn14 has an important role in the pathogenesis of systemic diseases such as lupus erythematosus^8–11^. Moreover, the TWEAK/Fn14 signals participate in the pathophysiological processes of a series of skin disorders including atopic dermatitis^12,13^, cutaneous vasculitis^14^, human papillomavirus infection^5^, and bullous pemphigoid^15^. In fact, TWEAK/Fn14 interaction modulates keratinocytes and dermal fibroblasts by enhancing cytokine production or collagen synthesis^1,13^. These findings clearly demonstrate that TWEAK/Fn14 activation is pivotal in the development of cutaneous inflammation.

Psoriasis is a chronic inflammatory skin disease with high prevalence^16^. Recent studies suggested that TWEAK/Fn14 signaling is involved in the pathogenesis of psoriasis^12,13,17^. It was originally found that Fn14 but not TWEAK is highly expressed in the lesional skin of patients with psoriasis^12^. However, more evidence supported that both TWEAK and Fn14 are upregulated in psoriatic lesion of patients or murine models^13,17–19^. The serum level of TWEAK is also elevated in patients with psoriasis vulgaris or psoriatic arthritis^20,21^. Moreover, Fn14 is highly expressed in lesional epidermis of patients with psoriasis, and the stimulation of psoriatic cytokines enhanced the Fn14 expression by keratinocytes in vitro^17^. TWEAK also enhances the proliferation of keratinocytes in vitro that are simultaneously stimulated with psoriatic cytokines^17^. TNF receptor (TNFR) type 2 is upregulated in psoriatic keratinocytes, and contributes to the TWEAK-induced keratinocyte proliferation^17^. In addition, TWEAK deficiency in mice results in defective maintenance of psoriasis-specific T helper type17 (Th17) cells in the skin, and impairs the expression of disease-characteristic cytokines such as chemokine (C-C motif) ligand 20 and interleukin-19 (IL-19) in a murine psoriasis-like model^13^. Therefore, TWEAK/Fn14 signals are activated in psoriasis and TWEAK is a critical contributor to psoriatic inflammation.

However, current findings merely suggest that TWEAK/Fn14 activation is prominent in psoriatic lesion and TWEAK inhibition attenuates psoriatic inflammation in the murine model. In fact, soluble TWEAK is synthesized by infiltrating immune cells and distributes in both sera and various tissues^10,15^. Fn14 is predominantly expressed by resident cells, and hence relatively more restricted to local inflammatory tissues. Moreover, it had been demonstrated that Fn14 deficiency can effectively inhibit local inflammation and significantly ameliorates skin injuries in systemic disease^3,22^. Therefore, the purpose of this study focuses on further elucidating the effect of Fn14 inhibition on the development of psoriasis, and revealing potential downstream mechanism relevant to TWEAK/Fn14 function in psoriatic inflammation.

## Results

### Fn14 deficiency ameliorates psoriasis-like lesion in mice

**Fig. 1:**
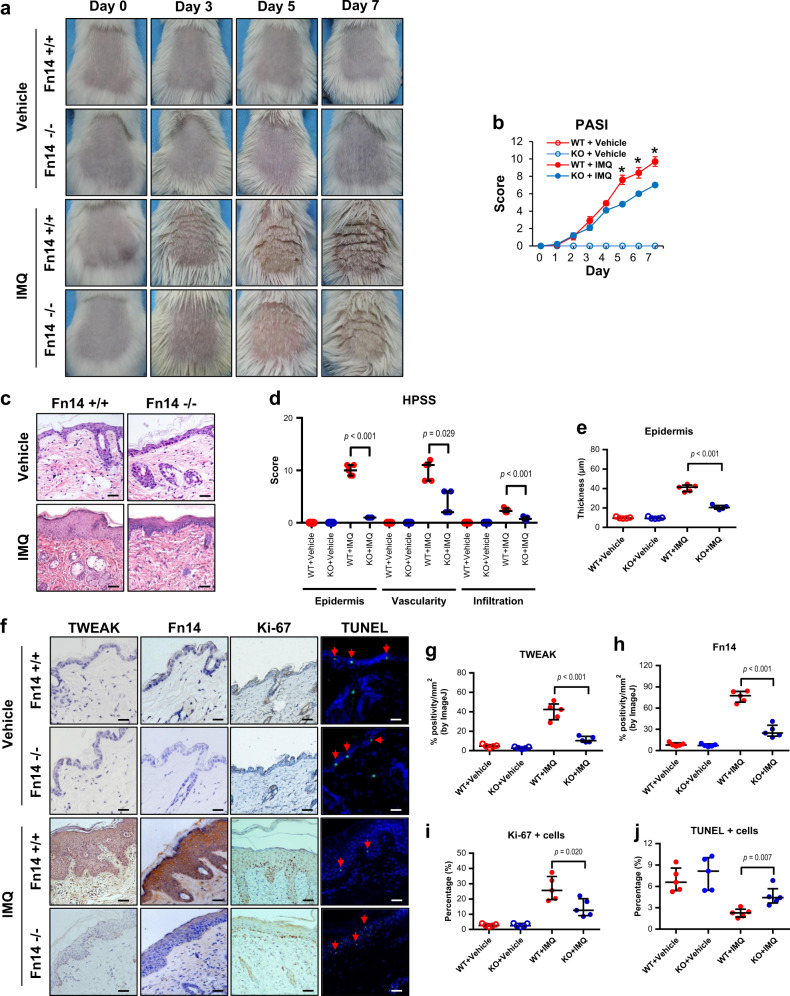
Fn14 deficiency dampens psoriasis-like skin disease in a murine model. Psoriasis-like disease was induced by daily application of 5% imiquimod (IMQ) cream to the shaved back of mice. The vehicle cream-treated mice were the controls. Skin tissue was harvested on day 7. **a** Psoriasis-like lesion was observed on days 0 to 7. **b** Skin lesion was evaluated for PASI scores; *p *<* 0.001, compared with the other groups. **c**, **d** The HE-stained sections were evaluated by HPSS method. The total HPSS scores were 22.35 ± 1.09 vs 5.40 ± 0.92 (IMQ-treated WT vs IMQ-treated KO), with *p* value < 0.001. **e** The epidermal thickness was measured on HE-stained sections. **f** By immunohistochemistry or TUNEL method, the sections were detected for the TWEAK and Fn14 expressions, Ki-67-positive cells, or apoptotic cells (green, indicated by arrowheads). **g**, **h** The epidermal intensities (% positivity) were quantitated by ImageJ software. **i**, **j** The percentages of Ki-67-positive or apoptotic cells were counted accordingly. Representative images are shown; *n* = 5 in each group. Bar = 20 μm. In (**b**, **d**, **e**, **g**, **h**, **i**, **j**), the IMQ-treated KO group had values different from the two vehicle groups since day 3 (*p* *<* 0.05)

Psoriasis-like lesion was monitored on the imiquimod-treated mice. The messenger RNA (mRNA) and protein expression levels of Fn14 increased with time in the imiquimod-treated wild-type (WT) strain (Supplementary Figure 1). It showed more severity in the WT strain as compared with the Fn14-knockout (KO) mice (Fig. 1a). Psoriasis area and severity index (PASI) was assessed accordingly, revealing higher scores in the WT mice during days 5 to 7 (Fig. 1b). Histopathological psoriasis severity score (HPSS) and epidermal thickness were evaluated with the hematoxylin–eosin (HE)-stained sections (day 7), and showed that the imiquimod-treated KO mice had less changes affecting the epidermis, blood vascularity, and inflammatory cells (Fig. 1c–e). By immunohistochemistry, the epidermal expression of TWEAK and Fn14 decreased in the imiquimod-treated KO mice on day 7 (Fig. 1f–h). Accordingly, the imiquimod-treated KO mice had less proliferating (Ki-67-positive) cells but more apoptotic cells in the epidermis (Fig. 1i, j). Both WT and KO mice exhibited no skin lesion after vehicle control treatment (Fig. 1).

### Fn14 deficiency reduces cutaneous infiltration of inflammatory cells in mice

**Fig. 2:**
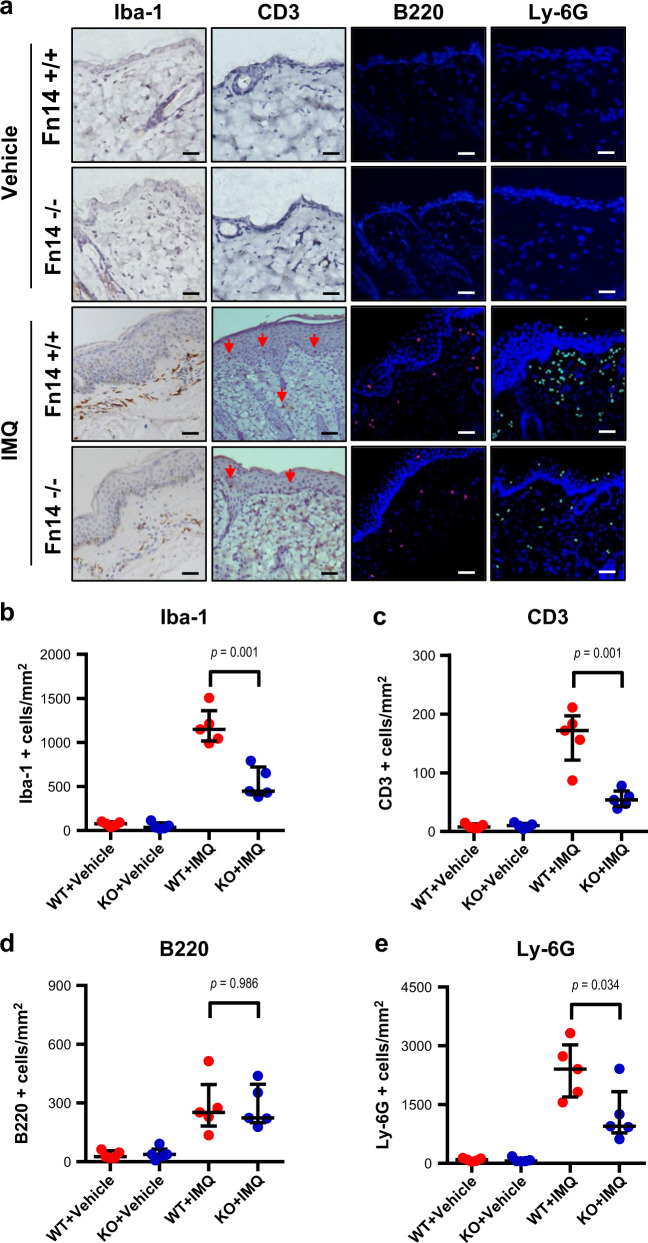
Fn14 deficiency reduces infiltration of inflammatory cells in psoriasis-like skin lesion. Psoriasis-like disease was induced by application of 5% imiquimod (IMQ) cream to the shaved back of mice. The vehicle cream-treated mice were the controls. Skin tissue was harvested on day 7. **a** By immunohistochemistry or immunofluorescent method, tissue sections were detected for Iba-1-, CD3-, B220 (red)-, or Ly-6G (green)-positive cells. The CD3-positive cells were indicated by arrowheads. **b**–**e** The positive cells were counted according to the areas of epidermis and dermis. Representative images are shown; *n* = 5 in each group. Bar = 20 μm. In (**b**–**e**), the two IMQ-treated groups had values higher than the two vehicle groups (*p* *<* 0.05)

Infiltration of inflammatory cells is central in the development of psoriasis, involving macrophages, T cells, B cells, and neutrophils^16,23–25^. By immunohistochemistry, we detected infiltrating cells in the skin on day 7. The Iba-1 (activated macrophages), CD3 (T lymphocytes), B220 (B lymphocytes), or Ly-6G (neutrophils) marker was used accordingly. It showed that the Fn14-KO mice had remarkably less Iba-1-, CD3-, or Ly-6G-positive cells in the skin lesion, while the distribution of B220-positive cells was similar between the two imiquimod-treated strains (Fig. 2a). The number of these cells was quantitated, verifying higher densities of Iba-1-, CD3- or Ly-6G-positive cells in the WT mice, and no significant difference in B220-positive cells between the two imiquimod-treated strains (Fig. 2b). There was no infiltration of inflammatory cells in the two vehicle control groups (Fig. 2).

### Proinflammatory cytokines are suppressed in the skin lesion of Fn14-deficient mice

**Fig. 3:**
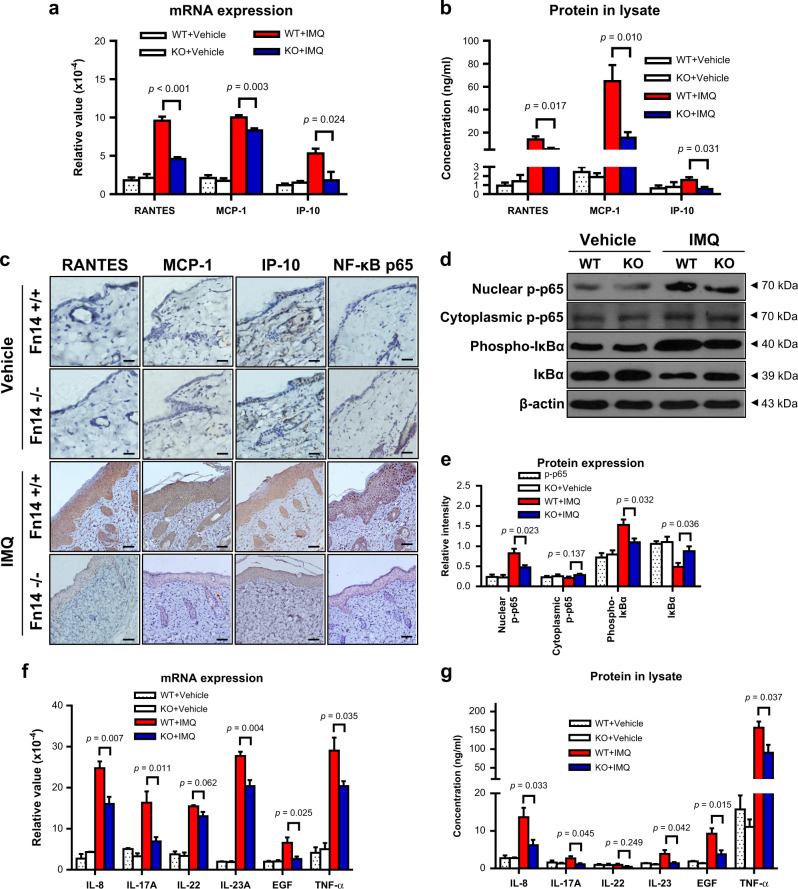
Fn14 deficiency attenuates the production of inflammatory cytokines in psoriasis-like skin disease. Psoriasis-like disease was induced by application of 5% imiquimod (IMQ) cream to the shaved back of mice. The vehicle cream-treated mice were the controls. Skin tissue was harvested on day 7. **a** The mRNA expression levels of RANTES, MCP-1, and IP-10 were determined in fresh tissue. **b** Accordingly, the proteins of RANTES, MCP-1, and IP-10 were determined by ELISA. **c** By immunohistochemistry, the sections were detected for the expressions of RANTES, MCP-1, IP-10, and NF-κB p65. **d**, **e** By western blotting, the NF-κB proteins were detected in the tissue lysates. **f** The mRNA expression levels of IL-8, IL-17A, IL-22, IL-23A, EGF, and TNF-α were determined in fresh tissue. **g** Accordingly, the proteins of IL-8, IL-17A, IL-22, IL-23, EGF, and TNF-α were determined by ELISA. In (**a**, **b**, **f**, **g**), the two IMQ-treated groups had values (except the protein of IP-10, IL-17A, IL-22 or IL-23 and the mRNA of IP-10 or EGF) higher than the two vehicle groups (*p* *<* 0.05). In (**e**), the IMQ-treated groups had higher values of nuclear p-p65 and phospho-IκB but lower value of IκB as compared with the vehicle groups (*p* *<* 0.05). p-p65 phospho-p65. Representative images are shown; *n* = 5 in each group. Bar = 20 μm

The expression of downstream cytokines was determined in these mice. It showed that on day 7, the imiquimod-treated KO mice had lower mRNA expression levels of RANTES (regulated upon activation normal T cell expressed and secreted), monocyte chemotactic protein-1 (MCP-1), and interferon-γ-induced protein 10 (IP-10) as compared with the imiquimod-treated WT mice (Fig. 3a). The protein levels of these cytokines were determined in tissue lysates by enzyme-linked immunosorbent assay (ELISA), also displaying lower expressions in the imiquimod-treated KO mice (Fig. 3b). These results were further mirrored by immunohistochemical staining and western blotting, which even showed a reduction of nuclear factor-κB (NF-κB) p65 expression in the imiquimod-treated KO mice (Fig. 3c–e). Other cytokines including IL-8, IL-17A, IL-22, IL-23, epidermal growth factor (EGF), and TNF-α were assessed in tissue samples, and exhibited lower expression levels (except IL-22) in the imiquimod-treated KO mice (Fig. 3f, g). The vehicle control groups had lower expression levels of most these cytokines as compared with the two imiquimod-treated groups (Fig. 3).

### Fn14 deficiency alters the TNFR and differentiation marker expression in mice

**Fig. 4:**
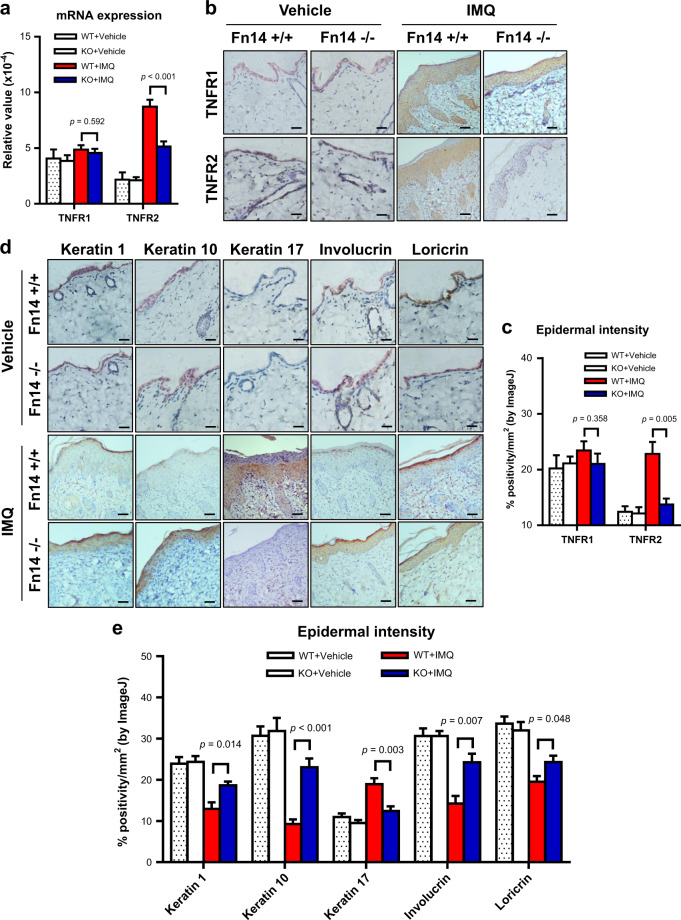
Fn14 deficiency modulates the TNFR and differentiation marker expression in psoriasis-like skin disease. Psoriasis-like disease was induced by application of 5% imiquimod (IMQ) cream to the shaved back of mice. The vehicle cream-treated mice were the controls. Skin tissue was harvested on day 7. **a** The mRNA expression levels of TNFR1 and TNFR2 were determined in fresh tissue. There was no significant difference in TNFR1 between different groups. **b**, **c** By immunohistochemistry, the sections were detected for the expression of TNFR1 and TNFR2. The epidermal intensities (% positivity) were quantitated by ImageJ software. **d**, **e** By immunohistochemistry, the sections were detected for the expressions of keratin 1, keratin 10, keratin 17, involucrin, and loricrin. The epidermal intensities were quantitated by ImageJ software. Representative images are shown; *n* = 5 in each group. Bar = 20 μm. In (**a**), the two IMQ-treated groups had higher TNFR2 values than the two vehicle groups (*p* *<* 0.05). In (**e**), the two IMQ-treated groups had values (except keratin 17) lower than the two vehicle groups (*p* *<* 0.05)

Recently, we demonstrated that the predominance of TNFR2 expression in keratinocytes accounts for the TWEAK/Fn14 signaling-induced keratinocyte proliferation^17^. In this study, we further evaluated the expression of both TNFR1 and TNFR2 in the skin lesion of the murine model. It showed that on day 7, the mRNA expression level of TNFR2 was higher in the imiquimod-treated WT mice, while there was no significant difference in the TNFR1 level among these groups (Fig. 4a). By immunohistochemistry, both the imiquimod-treated WT and KO mice had strong staining of TNFR1 in the epidermis (Fig. 4b). However, the TNFR2 staining was much stronger in the WT mice as compared with the KO mice after imiquimod treatment (Fig. 4b). The epidermal intensities (% positivity) were measured by ImageJ software, confirming less TNFR2 expression in the imiquimod-treated KO mice (Fig. 4c).

The differentiation markers including keratin 1, keratin 10, keratin 17, involucrin, and loricrin were determined in the skin tissues (day 7). The immunohistochemical results showed that their proteins (except keratin 17) were weaker in the WT mice as compared with KO mice after imiquimod treatment (Fig. 4d). Their expression levels were also quantitated in the epidermis, showing significant decrease in the imiquimod-treated WT mice (Fig. 4e). However, keratin 17 expression was more prominent in the imiquimod-treated WT mice (Fig. 4e).

### TWEAK exacerbates skin lesion in the WT but not in the Fn14-deficient mice

**Fig. 5:**
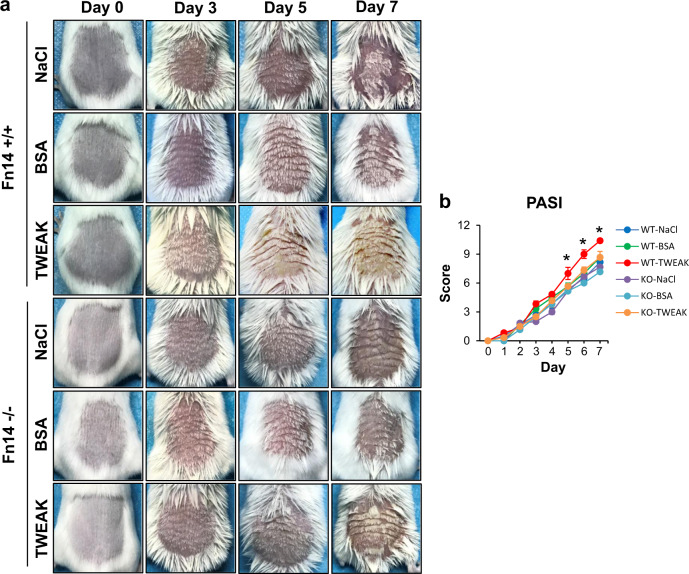
Topical treatment of TWEAK on the psoriasis-like skin disease in mice. Psoriasis-like disease was induced by imiquimod cream in the back of mice. Normal saline (NaCl), BSA, or TWEAK (20 µg/ml, 100 μl each time, twice daily) was also topically applied to skin lesion accordingly. Both BSA and TWEAK were dissolved in normal saline. **a** Representative images are shown. **b** Skin lesion was evaluated for PASI scores; *n* = 6 in each group. The TWEAK-treated WT mice had higher PASI scores than the other groups on days 5 to 7 (*p *<* 0.05). There were no significant differences between the TWEAK-treated KO mice and the NaCl- or BSA-treated KO mice (*p* *>* 0.05)

**Fig. 6:**
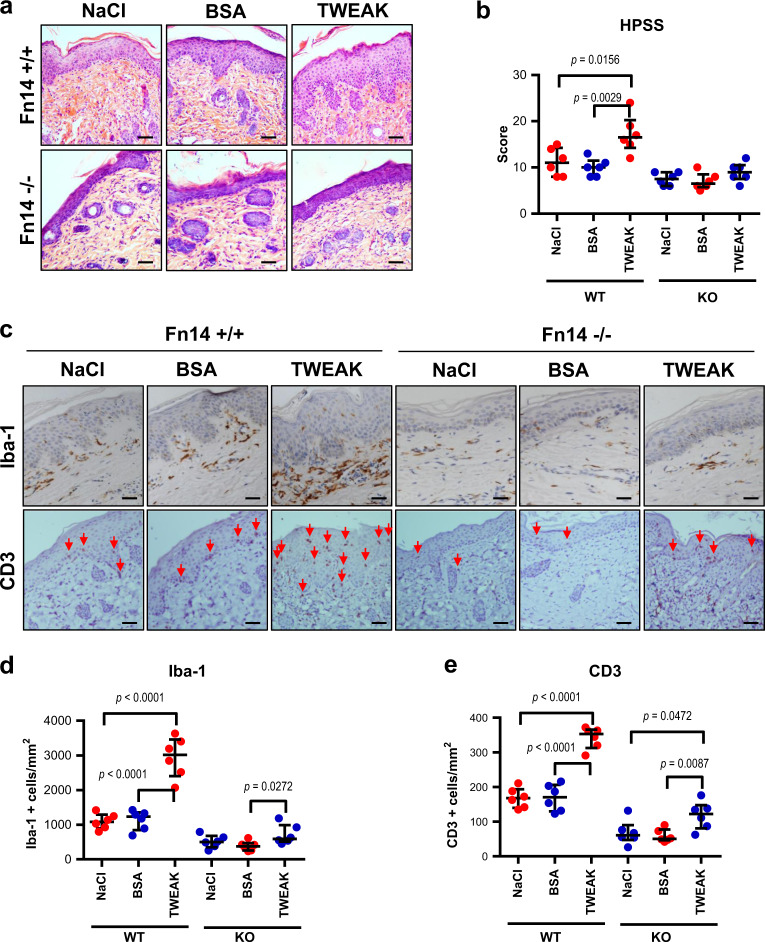
Exogenous TWEAK exacerbates psoriasis-like skin disease in mice. Psoriasis-like disease was induced by imiquimod cream in the back of mice. Recombinant TWEAK (20 µg/ml, 100 μl each time, twice daily) was also topically applied to skin lesion accordingly. Skin tissue was harvested on day 7. **a**, **b** The HE-stained sections were evaluated by HPSS method. There were no significant differences between the two control groups of the WT mice or between the three groups of the KO mice. **c** By immunohistochemistry, the sections were detected for the Iba-1- or CD3-positive cells. **d**, **e** The positive cells were counted according to the areas of epidermis and dermis. Representative images are shown; *n* = 6 in each group. Bar = 20 μm

The above results have demonstrated that Fn14 deficiency protects the murine model from psoriasis-like disease. We further investigated the effect of exogenous TWEAK on this murine model. It showed that in the WT mice, the topical application of recombinant TWEAK but not bovine serum albumin (BSA) exacerbated skin lesion and increased PASI scores as compared with the normal saline (NaCl) treatment (Fig. 5). However, both TWEAK and BSA exhibited no obvious effect on skin lesion in the Fn14-KO mice (Fig. 5). TWEAK alone induced neither visible nor histological change of skin in the non-imiquimod-treated WT mice (Supplementary Figure 2). Upon evaluation of HE-stained sections, the TWEAK-treated WT mice had higher HPSS score than the BSA- or NaCl-treated WT mice (Fig. 6a, b). There were no significant differences in HPSS score between these two controls of the WT mice or between the three groups of the KO mice (Fig. 6b). Tissue sections were also evaluated for Iba-1- or CD3-positive cells by immunohistochemistry. It showed that the TWEAK-treated WT mice had more intense infiltration of Iba-1- or CD3-positive cells in the skin lesion, while such difference was slight in the Fn14-KO mice (Fig. 6c). The number of Iba-1- or CD3-positive cells were counted, showing that the WT mice had more intense infiltration upon TWEAK application, while such effect of TWEAK was slight in the Fn14-KO mice (Fig. 6d, e).

### TWEAK suppresses differentiation but enhances EGFR expression of psoriatic keratinocytes

**Fig. 7:**
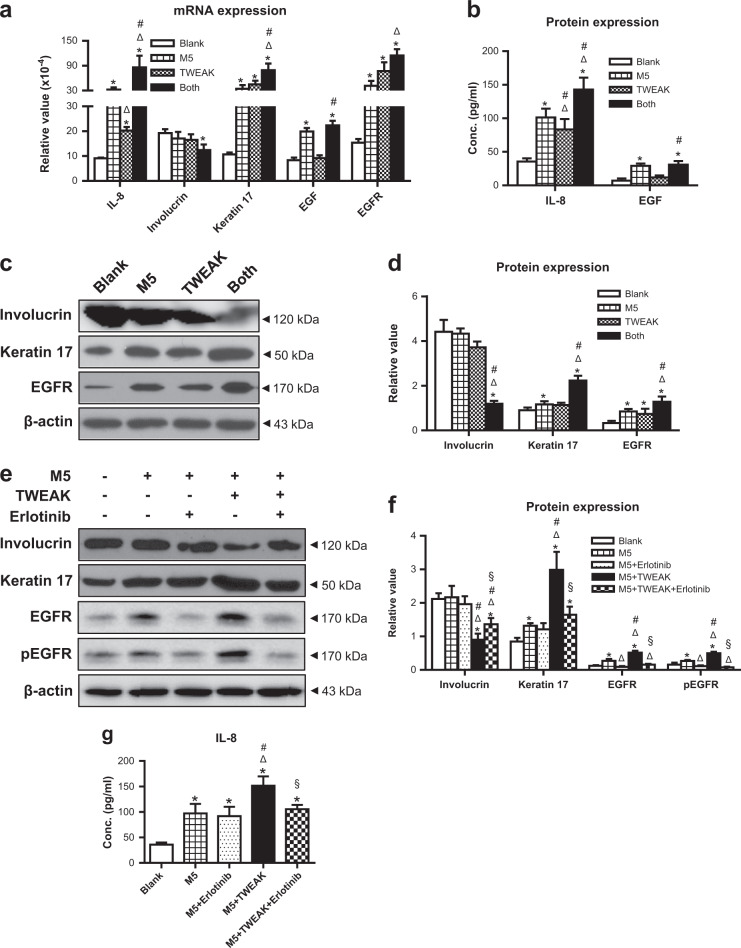
TWEAK enhances inflammatory responses but suppresses differentiation of psoriatic keratinocytes. Human primary keratinocytes were cultured in vitro with the 48 h addition of M5 cytokines (10 ng/ml each component), TWEAK (100 ng/ml), or erlotinib (10 µM). **a** The mRNA expression levels of IL-8, involucrin, keratin 17, EGF, and EGFR were determined in cell lysates. **b** Accordingly, the IL-8 and EGF proteins were assessed by ELISA in the culture supernatants. **c** By western blotting, involucrin, keratin 17, and EGFR were detected in the protein lysates of cell cultures. **d** The intensities of western blots were quantitated by ImageJ software. **e**, **f** By western blotting, the involucrin, keratin 17, EGFR, and phospho-EGFR (pEGFR) proteins were detected in the protein lysates. Their intensities were quantitated by ImageJ software accordingly. **g** By ELISA, IL-8 was assessed in the culture supernatants after erlotinib treatment. Representative images are shown. Data were obtained from three to five independent experiments. In (**a**, **b**, **d**), *p < 0.05, compared with the blank group; ^Δ^*p* < 0.05, compared with the M5 alone group; ^#^*p* < 0.05, compared with the TWEAK alone group. In (**f**, **g**), *p < 0.05, compared with the blank group; ^Δ^*p* < 0.05, compared with the M5 alone group; ^#^*p* < 0.05, compared with the M5 plus erlotinib group; ^§^*p* < 0.05, compared with the M5 plus TWEAK group

To further elucidate the effect of TWEAK/Fn14 signaling on keratinocytes under psoriatic inflammation, we constructed an in vitro psoriatic model by adding M5 cytokines (a cocktail of IL-1α, IL-17A, IL-22, oncostatin M, and TNF-α)^26^. The results showed that TWEAK alone increased the mRNA expression levels of IL-8, keratin 17, and epidermal growth factor receptor (EGFR) (Fig. 7a). Moreover, TWEAK and M5 exhibited synthetic effect on their mRNA expression (Fig. 7a). TWEAK or M5 alone did not affect the mRNA level of involucrin, while their combination suppressed such level (Fig. 7a). The proteins of these molecules were determined by ELISA or western blotting. It showed that TWEAK and M5 synergistically elevated the IL-8, keratin 17, and EGFR expression but inhibited involucrin expression (Fig. 7b–d). The mRNA and protein expression levels of EGF were enhanced by M5 but not by TWEAK alone (Fig. 7a, b).

**Fig. 8:**
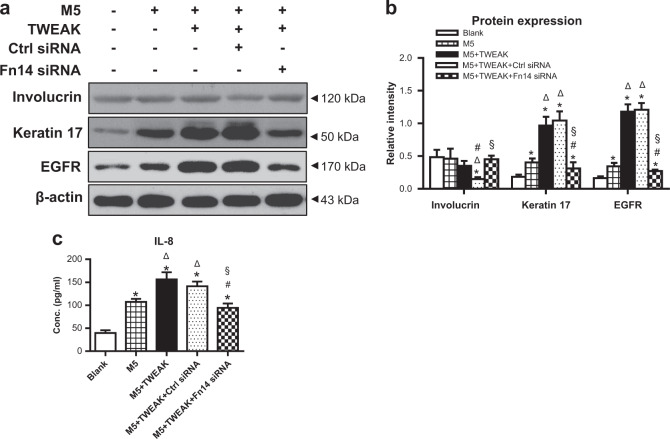
Fn14 siRNA transfection abrogated the TWEAK effect on psoriatic keratinocytes. Human primary keratinocytes were cultured in vitro with the 48 h addition of M5 cytokines (10 ng/ml each component) or plus TWEAK (100 ng/ml). Some cells were pretreated with siRNA transfection. **a** By western blotting, the involucrin, keratin 17, and EGFR proteins were detected in the lysates. **b** The intensities of western blots were measured by ImageJ software. **c** By ELISA, IL-8 was assessed in the culture supernatants after different treatments. Representative images are shown. Data were obtained from three independent experiments; *p < 0.05, compared with the blank group; ^Δ^*p* < 0.05, compared with the M5 alone group; ^#^*p* < 0.05, compared with the M5 plus TWEAK group; ^§^*p* < 0.05, compared with the M5, TWEAK plus control siRNA group

Previous study demonstrated that EGFR signaling mediates the function of TWEAK in extracutaneous inflammation^27^. Here, we pretreated these psoriatic keratinocytes with a specific inhibitor (erlotinib) of EGFR. It showed that erlotinib abrogated the effect of TWEAK on the expressions of involucrin, keratin 17, EGFR, and phospho-EGFR in keratinocytes (Fig. 7e, f). TWEAK-induced IL-8 upregulation was also abrogated in these cells (Fig. 7g). Similarly, the effect of TWEAK on the involucrin, keratin 17, EGFR, and IL-8 expressions diminished upon Fn14 small interfering RNA (siRNA) transfection, which did not influence the effect of M5 cytokines (Fig. 8). Obviously, the receptors EGFR and Fn14 mediated TWEAK effect on keratinocytes under psoriatic inflammation.

### TWEAK enhances the proliferation and IL-6 production of dermal microvascular endothelial cells (DMECs) under psoriatic inflammation

The effect of TWEAK/Fn14 activation on DMECs was also studied by growing cells with the addition of M5 cytokines. The results showed that DMECs exhibited higher proliferation ratio after TWEAK stimulation. Moreover, TWEAK elevated the mRNA expression level of IL-6 and the IL-6 concentration in culture supernatants. However, such effect of TWEAK on DMECs was abrogated by the transfection of Fn14 siRNA. TWEAK exhibited no effect on the apoptosis of these psoriatic DMECs (*p* > 0.05). See Supplementary Figure 3.

## Discussion

In this study, we demonstrated that Fn14 deficiency significantly ameliorates psoriasis-like disease in a murine model. The attenuation of skin lesion involves the tempered inflammatory cell infiltration and proinflammatory cytokine production in local tissue. Accordingly, the TNFR2 expression decreases in the lesional epidermis of the Fn14-deficient mice. Moreover, exogenous TWEAK exacerbates skin lesion in normal but not in Fn14-deficient mice. In addition, TWEAK enhances inflammatory responses but suppresses differentiation of psoriatic keratinocytes in vitro. Inhibition of EGFR or Fn14 can abrogate the TWEAK effect on these keratinocytes. Therefore, the TWEAK/Fn14 signals participate in the development of psoriatic skin disease in mice.

Previous studies had demonstrated that the TWEAK/Fn14 signals are activated in the skin lesion of psoriasis, and downstream proinflammatory cytokines are produced consequently^28^. Recently, we found that TWEAK/Fn14 interaction promotes the proliferation of keratinocytes under M5-induced psoriatic inflammation^17^. Sidler et al.^13^ found that subcutaneous injection of TWEAK into naive mice induces cutaneous inflammation with histological and molecular signs of psoriasis, which can be abrogated by Fn14 deficiency. On the other hand, TWEAK deficiency attenuates histological changes in imiquimod-induced murine model^13^. In this study, we directly observed imiquimod-induced psoriasis-like disease in Fn14-deficient mice. Moreover, we topically administrated TWEAK solution to the skin lesion in this model. Our results clearly showed that Fn14 deficiency ameliorates psoriasis-like disease while topical TWEAK application exacerbates the skin lesion in this model. The difference in PASI or HPSS score between the WT and KO strains became less obvious after such solution administration. This phenomenon could be explained that a solution treatment may reduce the scale formation in mice. Since Fn14 upregulation is relatively restricted to injured tissue, inhibition of Fn14 but not TWEAK might be more specific in reducing psoriatic lesion. We found that topical application of TWEAK solution induces no psoriasis-like phenotype in normal skin. This may be due to the limited permeability of TWEAK solution on the intact skin. Therefore, our findings provided stronger evidence supporting that targeting the TWEAK/Fn14 pathway might be promising in the treatment of psoriasis.

Previously, it was found that inflammatory cells including macrophages, T cells, and neutrophils participate in the pathogenesis of psoriasis^16,23,25^. These inflammatory cells may secret proinflammatory cytokines such as soluble TWEAK^28^, and also growth factors including EGF^29^. In this study, we found that Iba-1-, CD3-, or Ly-6G-positive cells infiltrate intensely in the epidermis or dermis in psoriatic lesion. The Fn14 deficiency-related attenuation and additional TWEAK-related exacerbation of psoriatic lesion also correlate positively with the alteration of their infiltrate. B cells have been suggested to be involved in the pathogenesis of psoriasis^24^. However, the role of B cells in psoriasis depends on their subtypes. For example, regulatory B cells especially those producing IL-10 can suppress psoriasis-like skin inflammation in imiquimod-induced model^24^. Moreover, B cells are hardly detected in psoriatic skin^30^. In this study, the distribution of B220-positive cells is scarce in lesional skin and does not alter upon Fn14 deficiency. Therefore, it is worth investigating the effect of Fn14 inhibition on different types of B cells in further studies.

### Many proinflammatory cytokines participate in the processes of psoriatic inflammation

RANTES, IP-10, MCP-1, and IL-8 not only accumulate in psoriatic skin, but also correlate with the TWEAK/Fn14 activation^17^. Psoriasis induces a Th1 inflammatory response at the affected site, leading to overproduction of TNF-α, IL-17, and IL-23^17^. In this study, we found that these cytokines (except IL-22) are suppressed in the skin lesion of Fn14-deficeint mice. Although IL-22 promotes the expression of Fn14 in keratinocytes^17^, TWEAK/Fn14 activation may not affect the IL-22 production in a feed-back manner. Hence, IL-22 expression is not fluctuated in this model upon Fn14 inhibition.

The abundant TNF-α in psoriatic lesion acts by binding to its receptor, which has two subtypes, TNFR1 and TNFR2. Recently, we found that TNFR2 is predominantly expressed in keratinocytes under certain inflammatory microenvironments including psoriasis^5,17^. TWEAK/Fn14 interaction induces the proliferation but not apoptosis of keratinocytes that have TNFR2 predominance^5,17^. Actually, an Fn14–TRAF2 (TNFR-associated factor type 2)–TNFR axis mediates the functional directions of TWEAK/Fn14 signaling^31^. In this study, we found that both TNFR1 and TNFR2 are highly expressed in psoriatic lesion in the WT mice. However, the TNFR2 expression decreases more obviously in the Fn14-deficient mice. Since TNFR2 activation antagonizes TNFR1-induced caspase signaling^17^, such alteration of the TNFR1/TNFR2 expression profile may generate cell-fate diversity of keratinocytes, reflecting an effect of the TWEAK/Fn14 signals on psoriatic epidermis.

The poor differentiation of keratinocytes can reflect the pathological changes of psoriatic skin. We found that the differentiation markers, including keratin 1, keratin 10, involucrin, and loricrin, are more expressed in Fn14-deficient mice as compared with the WT mice. This further confirmed that Fn14 inhibition ameliorates psoriatic changes in this model. However, keratin 17 expression decreases in the Fn14-deficient model. In fact, keratin 17 is upregulated in psoriatic keratinocytes and contributes to the development of this disease^32^. Therefore, Fn14 inhibition downregulates keratin 17 expression consistently.

EGF participates in the pathogenesis of psoriasis through modulating keratinocyte proliferation, angiogenesis, and cell differentiation in the epidermis^33^. Its receptor, EGFR, is overexpressed in psoriatic lesions, and correlates with the hyperproliferation of keratinocytes in psoriasis^34^. Recent study demonstrated that EGFR signaling mediates the function of TWEAK in renal inflammation, leading to downstream effects including extracellular signal–regulated kinase activation^27^. We found that EGF expression is attenuated in the murine model upon Fn14 deficiency. Also, both M5 cytokines and TWEAK upregulate EGFR expression in keratinocytes in vitro. These findings demonstrated that EGFR signaling fluctuates with TWEAK/Fn14 activation during the development of psoriasis. An interesting phenomenon was that inhibition of EGFR (by erlotinib) significantly reduces the effect of TWEAK on psoriatic keratinocytes. Obviously, EGFR signaling mediates the role of TWEAK/Fn14 signals in psoriatic lesion. In fact, blocking EGFR signals has been suggested in the treatment of psoriasis vulgaris^35^. Therefore, the EGFR signaling pathway should be considered in further elucidation of the TWEAK/Fn14 signals in psoriatic inflammation.

M5 cytokines can induce inflammatory responses in skin or keratinocytes recapitulating some features of psoriasis^36^. Keratinocyte culture under M5 stimulation has been used as an in vitro model of psoriasis^17,26^. In this study, the changes of differentiation markers (involucrin and keratin 17) and inflammatory cytokine (IL-8) were consistent with that in psoriatic tissue. Keratin 17 not only reflects the differentiation status of epidermal keratinocytes, but also participates in the pathogenesis of psoriasis by interacting with IL-17A and IL-22^32^. We also found that M5 cytokines and TWEAK/Fn14 signaling exhibit an effect on keratin 17 expression in keratinocytes. Hence, TWEAK/Fn14 signaling functions through regulating keratin 17-related inflammation.

IL-6 can be produced by DMECs, and contributes to psoriatic inflammation through participating in Th17 cell differentiation^37^. Moreover, TWEAK acts on DMECs through inducing rapid phosphorylation of inhibitor of κB-α and then regulating cytokine production^14^. In this study, we observed that TWEAK enhances the proliferation and IL-6 production of DMECs under psoriatic inflammation. This finding further demonstrated that TWEAK/Fn14 activation participates in the development of psoriasis involving the modulation of resident cells other than keratinocytes.

In conclusion, Fn14 deficiency ameliorates psoriasis-like lesion in a murine model, involving the suppression of inflammatory responses in the skin lesion. Moreover, topical application of TWEAK amplifies psoriatic lesion in this model. TWEAK/Fn14 signaling contributes to the development of psoriasis by upregulating the TNFR2, EGFR, and keratin 17 expressions in keratinocytes and the IL-6 expression in DMECs. Targeting the TWEAK/Fn14 pathway may be a promising therapeutic approach in the management of patients with psoriasis.

## Materials and methods

### Murine model

Fn14 deficiency was generated in BALB/c mice by using the clustered, regularly interspaced, short palindromic repeats (CRISPR)/CRISPR-associated (Cas) 9 method^38^. Fn14−/− homozygote mice were selected after the hybridization of F0 generation mice. The efficiency of Fn14 deficiency was confirmed by both polymerase chain reaction (PCR) and western blotting (data not shown). The CRISPR/Cas9 engineering and reproduction were conducted in the Shanghai Biomodel Organism Science and Technology Company (Shanghai, China; Protocol No. N1–861). The serum levels of total IgG and the distributions of CD4-, CD8-, B220-, or Ly-6G-positive cells in the spleens were determined, showing no significant differences between the WT and KO strains (Supplementary Figure 4). Mice were housed at the Medical Animal Center of Xi’an Jiaotong University. Male mice at age of 10 weeks were used in the experiments. This study was performed under the supervision of the Hospital Research Ethics Committee.

To induce psoriasis-like skin disease, 5% imiquimod cream (or vehicle control; Sichuan Mingxin Pharmaceuticals Co., China) was applied daily to the shaved back of mice^13^. The topical dose of creams was 25 mg daily, translating in a dose of 1.25 mg of imiquimod. In some experiments, recombinant murine TWEAK (R&D Systems, Minneapolis, MN, USA) or BSA (Sigma-Aldrich, St Louis, MO) was prepared in normal saline (20 µg/ml), and then twice daily (100 μl each time) administrated to the surface of the skin lesion. The TWEAK administration was performed 4 h after imiquimod treatment. Mice were monitored for PASI scores by two dermatologists blinded to the grouping^39^. Skin tissues were collected on day 7.

### Immunohistochemistry and histological evaluation

Immunohistochemistry was performed as described previously^40^. In brief, paraffin sections were deparaffinized and rehydrated, and then blocked with Dual Endogenous Enzyme Block (DAKO, Glostrup, Denmark). Rabbit anti-TWEAK (or Fn14, Ki-67, Iba-1, CD3, RANTES, MCP-1, IP-10, NF-κB p65, keratin 1, keratin 10, keratin 17, TNFR1, TNFR2, involucrin, or loricrin) IgG (2 µg/ml; Abcam, Cambridge, MA) was used as primary antibody. Polymer-horseradish peroxidase-labeled goat anti-rabbit IgG was the secondary antibody (DAKO). Some sections were stained with isotype control antibodies (Abcam, Cambridge, MA) (Supplementary Figure 5). Sections were then incubated with 3, 3′-diaminobenzine-chromogen substrate (DAKO), followed by HE counterstaining.

HPSS scoring was performed with HE-stained sections. HPSS scoring grades for overall epidermis, vascular, and infiltrate changes^41^. The epidermal score include the changes of thickness, parakeratosis, granular layer, rete ridges, dermal papillae, and other relevant architectures^42^. The percentage of Ki-67-positive cells was counted in the epidermis. The number of Iba-1- or CD3-positive cells was counted in both epidermal and dermal areas. On the keratin 1, keratin 10, keratin 17, TNFR1, TNFR2, involucrin, or loricrin-stained sections, the positively stained epidermis was quantified by ImageJ software (National Institutes of Health, Bethesda, MD, USA) according to a previous method^15^. One representative section of each mouse and five fields in each section were selected randomly for histological evaluation. Such evaluation was performed in a blinded fashion by two pathologists.

### Immunofluorescence

Immunofluorescence was carried out with frozen sections^11^. Briefly, sections were fixed with pre-cooled (−20 °C) acetone and then blocked with 10% BSA in phosphate-buffered saline. Phycoerythrin-conjugated rat anti-B220 IgG or fluorescein isothiocyanate-conjugated rat anti-Ly-6G IgG (Abcam) was used as primary antibodies (2 µg/ml). Before observation under digital confocal microscopy (Leica, Wetzlar, Germany), sections were counterstained with 4′,6-diamidino-2-phenylindole. Terminal deoxynucleotidyl transferase dUTP nick end labeling (TUNEL; Beyotime Biotech, Beijing, China) was used for detecting apoptotic cells. The number of positive cells in both epidermal and dermal areas or the percentages of apoptotic cells in epidermis were calculated accordingly.

### Cell culture

Human primary keratinocytes (Life Technologies, Carlsbad, CA) were cultured in EpiLife® medium supplemented with Supplement S7 (Life Technologies). Before stimulation assays, keratinocytes were routinely starved in non-fetal bovine serum-supplemented Dulbecco’s modified Eagle’s medium for 24 h. Then, cell cultures were added with M5 cocktail (IL-1α, IL-17A, IL-22, oncostatin M, and TNF-α, each at 10 ng/ml) or TWEAK (100 ng/ml) for 48 h. Such duration and concentration of TWEAK are efficient for inducing inflammatory in normal keratinocytes or proliferative responses in M5-treated keratinocytes^11,15,17^. In some experiments, the EGFR inhibitor erlotinib (10 µM; Sigma-Aldrich) was added to the culture media simultaneously. Primary human DMECs (Life Technologies) were routinely cultured in RPMI 1640 media. Similarly, DMECs were also treated with M5 cocktail or plus TWEAK (100 ng/ml) for 48 h.

Some cells were transfected with Fn14 or control siRNA before stimulation assays^15^. The siRNA oligonucleotides were mixed with Lipofectamine 2000 transfection reagent (75 pmol siRNA: 7.5 µl of reagent; Life Technologies), and then added to subconfluent cells in 6-well plates for 24 h. The transfection efficiency was verified by both quantitative real-time PCR (qRT-PCR) and western blotting (Supplementary Figure 6).

### Proliferation and apoptosis assays

Proliferation and apoptosis assays were performed on DMECs as described previously^5^. Cell proliferation and apoptosis were determined with CellTiter 96® Solution (Promega, Madison, WI) and the Muse™ Caspase-3/7 Kit (Millipore, Billerica, MA), respectively.

### ELISAs

ELISAs were performed with both culture supernatants and tissue lysates. Fresh tissue was processed for protein lysates, which were then dissolved in phosphate-buffered saline. All lysate samples were normalized according to the weight of the original tissue. The commercial immunoassay kits (targeting mouse RANTES, MCP-1, IP-10, IL-6, IL-17A, IL-22, IL-23, EGF or TNF-α or targeting human IL-8 or EGF) were purchased from R&D Systems. The commercial immunoassay kit targeting mouse IL-8 was purchased from YL Biotech Co. (Shanghai, China). The protocols were provided by the manufacturers.

### qRT-PCR

Fresh tissue and cell cultures were processed for total RNA extraction by using the Trizol reagent (Ambion, Carlsbad, CA). Reverse transcription was performed for complementary DNA by using a commercial kit (Takara Bio, Kyoto, Japan). qRT-PCR was carried out on the 7900HT Fast PCR System (Applied Biosystems, Waltham, MA). SYBR Green Master Mixes (Takara Bio) was used as fluorescent dye. The PCR primers were synthesized by AuGCT DNA-SYN Biotech (Beijing, China), and listed in Supplementary Table 1. The expression levels (relative values) of the objective genes were calculated using the 2^−ΔCt^ method. Glyceraldehyde 3-phosphate dehydrogenase gene was the control.

### Western blotting

Protein lysates were extracted from fresh tissue and cultures. They were separated on electrophoresis gels, followed by transfer onto polyvinylidene difluoride membrane (Millipore, Billerica, MA). Rabbit antibodies to keratin 17, EGFR, involucrin, phospho-p65 or β-actin (2 µg/ml; Abcam), rabbit antibodies to phospho-EGFR (Tyr845), phospho-IκBα or IκBα (2 µg/ml; Cell Signaling, Danvers, MA), and horseradish peroxidase-conjugated goat anti-rabbit IgG (1 µg/ml; Abcam) were used as the primary antibodies and secondary antibody, respectively. Signal was developed using an ECL chemiluminescence kit (Millipore). The band intensities were quantitated using ImageJ software and normalized to β-actin values accordingly.

### Statistical analysis

All data were expressed as mean ± standard error of mean. Statistical analysis was performed by using GraphPad Prism version 5.0 (GraphPad Software, La Jolla, CA). Analysis of variance was used for the comparison of more than two groups. Then, a two-tailed Student’s *t*-test was used for statistical differences in comparing two groups. Differences were considered statistically significant at *p* < 0.05.

## Electronic supplementary material

supplementary data
