# Correction: Fn14 deficiency ameliorates psoriasis-like skin disease in a murine model

**DOI:** 10.1038/s41419-023-05758-4

**Published:** 2023-03-28

**Authors:** L. Peng, Q. Li, H. Wang, J. Wu, C. Li, Y. Liu, J. Liu, L. Xia, Y. Xia

**Affiliations:** 1 Department of Dermatology, The Second Affiliated Hospital, School of Medicine, Xi’an Jiaotong University, Xi’an, China; 2 Core Research Laboratory, The Second Affiliated Hospital, School of Medicine, Xi’an Jiaotong University, Xi’an, China

Correction to: *Cell Death and Disease* 10.1038/s41419-018-0820-6, published online 23 July 2018

The original version of this article contained errors in figures 3 and 4. The authors state the following: “In our experiments, the skin tissues from the Fn14-knockout and wild-type mice were somehow similar. Both immunohistochemistry and image collection were performed simultaneously for the vehicle-treated mice. Two images in Figures 3c (p65 staining) and 4d (keratin 10 staining) were generated from the sections of a knockout mouse. This mouse was treated with vehicle and its sections were labeled as wild-type ones due to negligence. Moreover, the differences in coloring were due to different staining by using those sections. For example, p65 staining was actually weak while keratin 10 was positive expressed in all vehicle-treated mice. The blue dashed box in Figure 3c differed from that in Figure 4d because of tissue loss during the antigen retrieval or buffer washing. The serial sections of single paraffin block brought some differences in dimensions of the images. We have replaced those images in Figures 3c and 4d (with boxes) with the correct ones. We believe that these mistakes do not affect the conclusions in this study. The corrected images can be found below.

**Figure:**
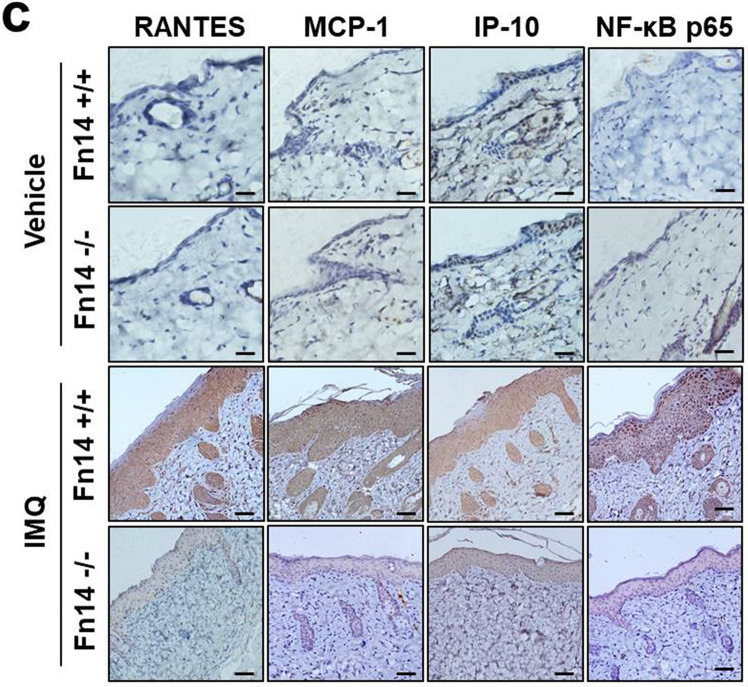
Fig. 3c

**Figure:**
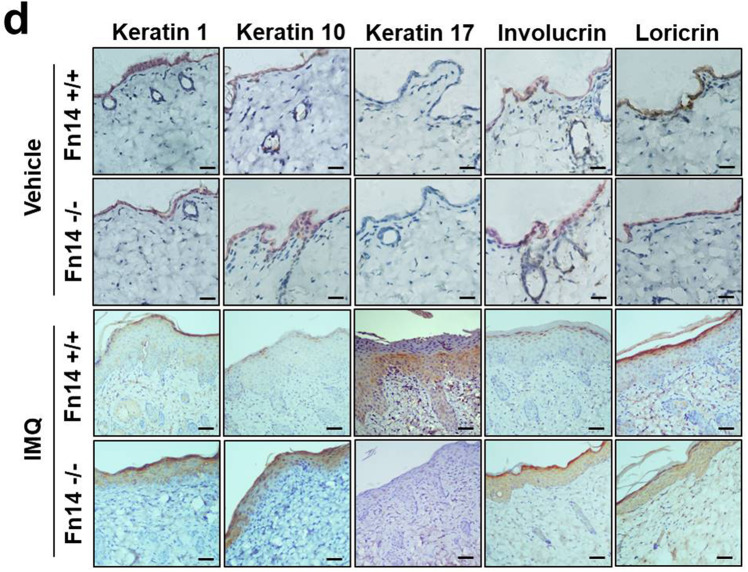
Fig. 4d

