# Supplementary material for: Fn14 deficiency ameliorates psoriasis-like skin disease in a murine model
Source: Cell Death Dis. 2018 Jul 23;9(8):801. doi: 10.1038/s41419-018-0820-6 (PMC6056551; doi:10.1038/s41419-018-0820-6)
Supplement: Supplementary file 1 — supplementary data [file 41419_2018_820_MOESM1_ESM.pptx]

## Slide 1
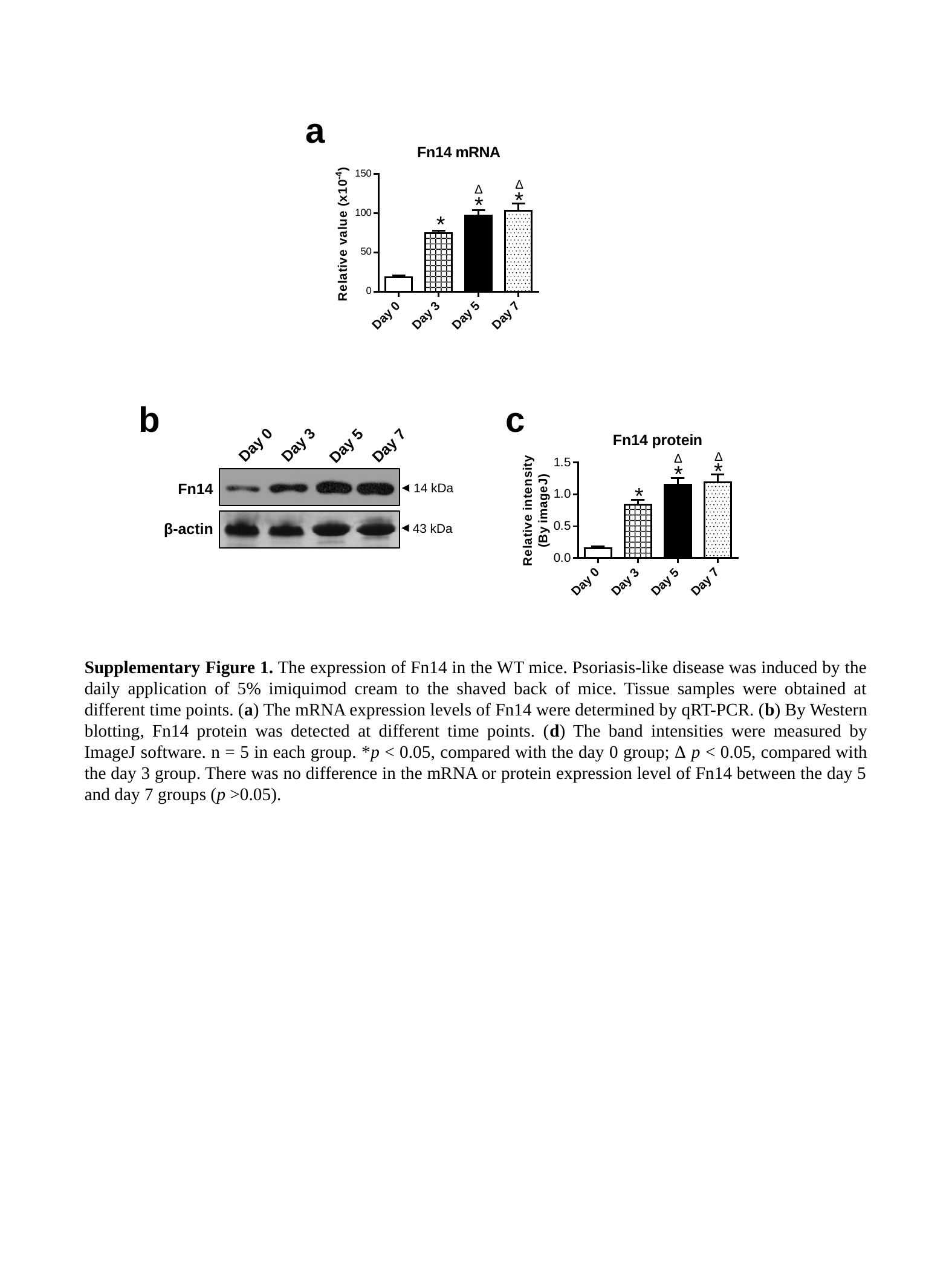

a
b
c
Day 3
Day 0
Day 7
Day 5
Fn14
14 kDa
β-actin
43 kDa
Supplementary Figure 1. The expression of Fn14 in the WT mice. Psoriasis-like disease was induced by the daily application of 5% imiquimod cream to the shaved back of mice. Tissue samples were obtained at different time points. (a) The mRNA expression levels of Fn14 were determined by qRT-PCR. (b) By Western blotting, Fn14 protein was detected at different time points. (d) The band intensities were measured by ImageJ software. n = 5 in each group. *p < 0.05, compared with the day 0 group; Δ p < 0.05, compared with the day 3 group. There was no difference in the mRNA or protein expression level of Fn14 between the day 5 and day 7 groups (p >0.05).

## Slide 2
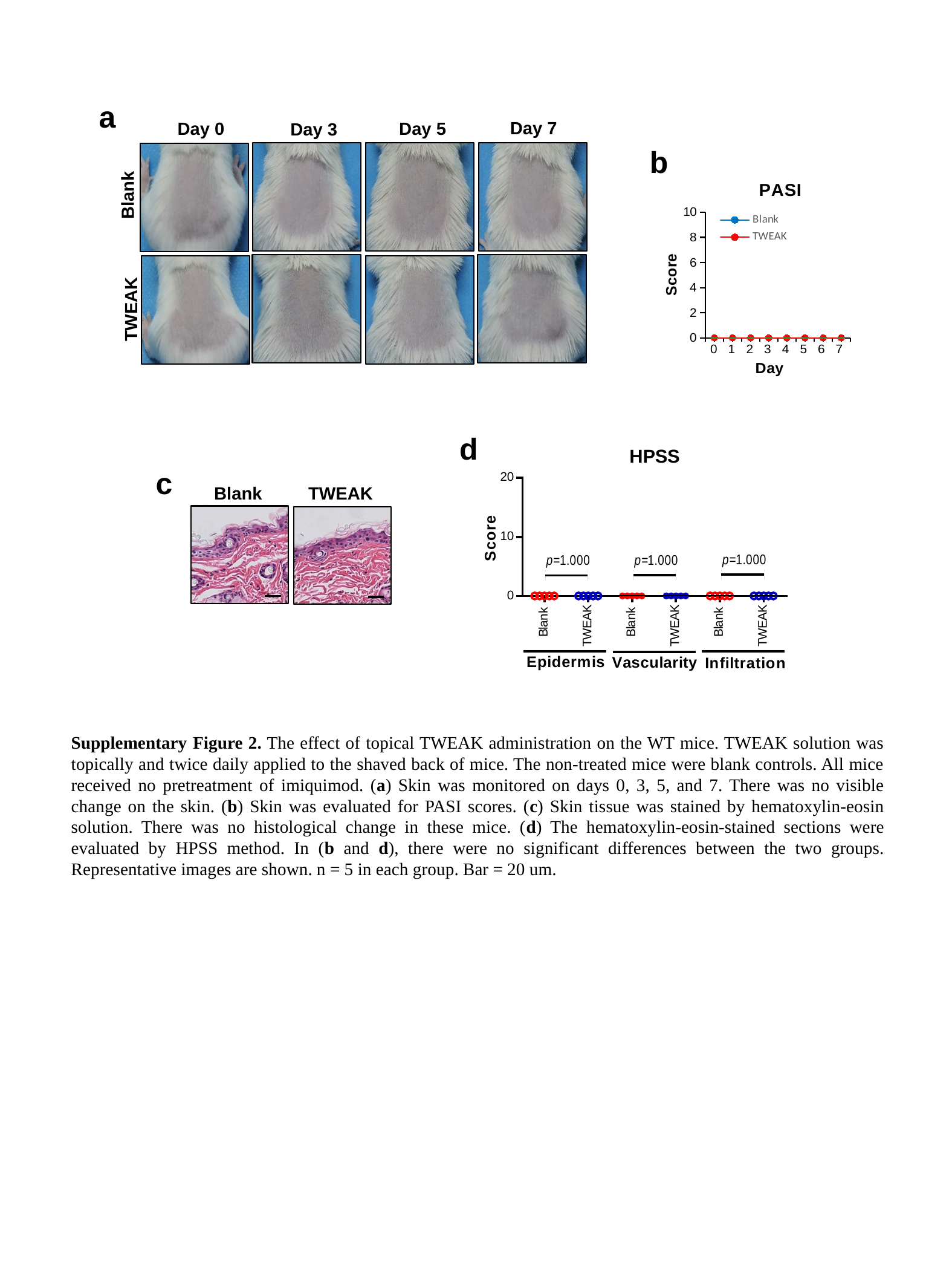

a
Day 7
Day 0
Day 5
Day 3
Blank
TWEAK
b
### Chart: PASI
| Category | | |
|---|---|---|d
c
Blank
TWEAK
Supplementary Figure 2. The effect of topical TWEAK administration on the WT mice. TWEAK solution was topically and twice daily applied to the shaved back of mice. The non-treated mice were blank controls. All mice received no pretreatment of imiquimod. (a) Skin was monitored on days 0, 3, 5, and 7. There was no visible change on the skin. (b) Skin was evaluated for PASI scores. (c) Skin tissue was stained by hematoxylin-eosin solution. There was no histological change in these mice. (d) The hematoxylin-eosin-stained sections were evaluated by HPSS method. In (b and d), there were no significant differences between the two groups. Representative images are shown. n = 5 in each group. Bar = 20 um.

## Slide 3
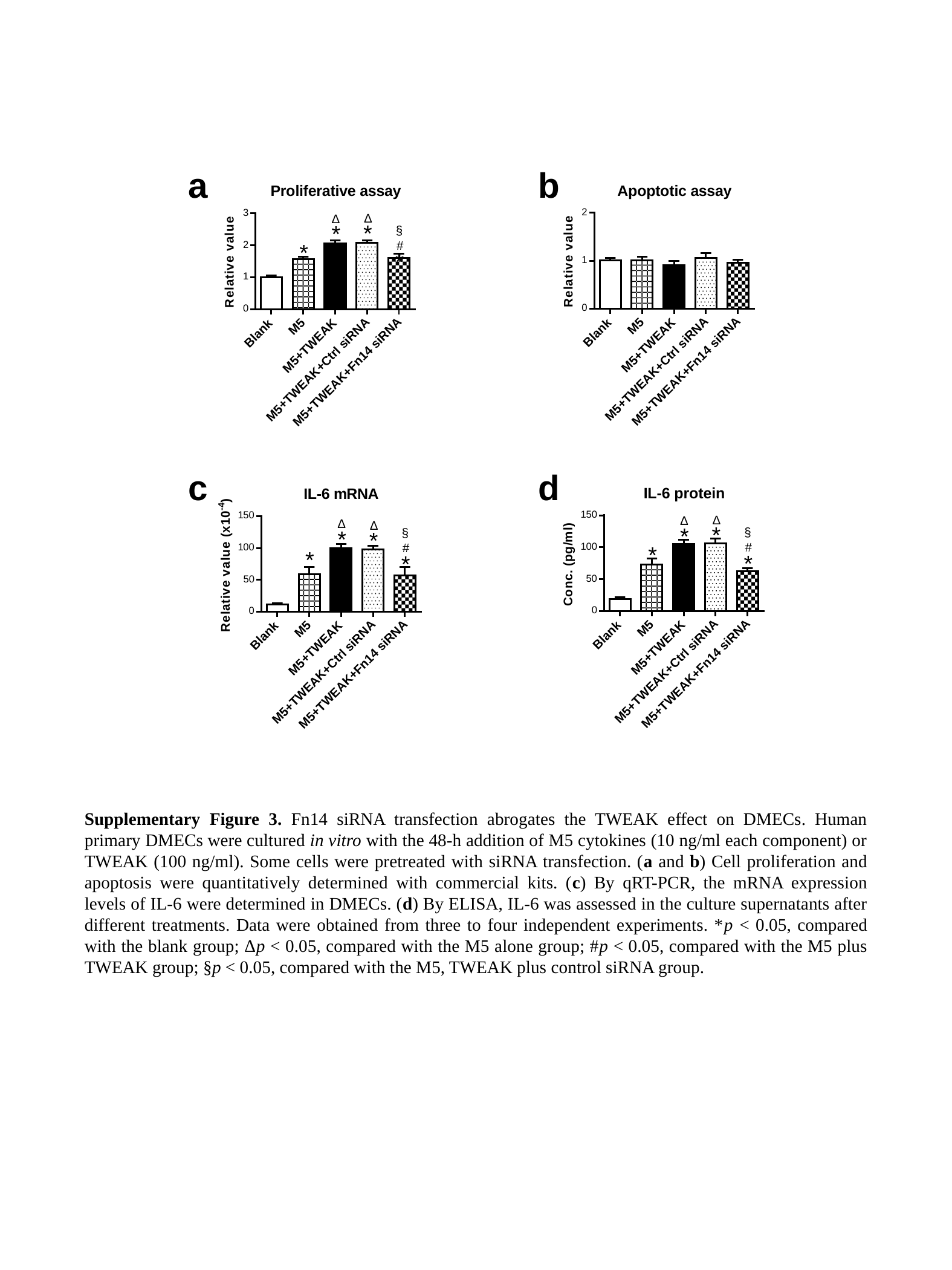

a
b
c
d
Supplementary Figure 3. Fn14 siRNA transfection abrogates the TWEAK effect on DMECs. Human primary DMECs were cultured in vitro with the 48-h addition of M5 cytokines (10 ng/ml each component) or TWEAK (100 ng/ml). Some cells were pretreated with siRNA transfection. (a and b) Cell proliferation and apoptosis were quantitatively determined with commercial kits. (c) By qRT-PCR, the mRNA expression levels of IL-6 were determined in DMECs. (d) By ELISA, IL-6 was assessed in the culture supernatants after different treatments. Data were obtained from three to four independent experiments. *p < 0.05, compared with the blank group; Δp < 0.05, compared with the M5 alone group; #p < 0.05, compared with the M5 plus TWEAK group; §p < 0.05, compared with the M5, TWEAK plus control siRNA group.

## Slide 4
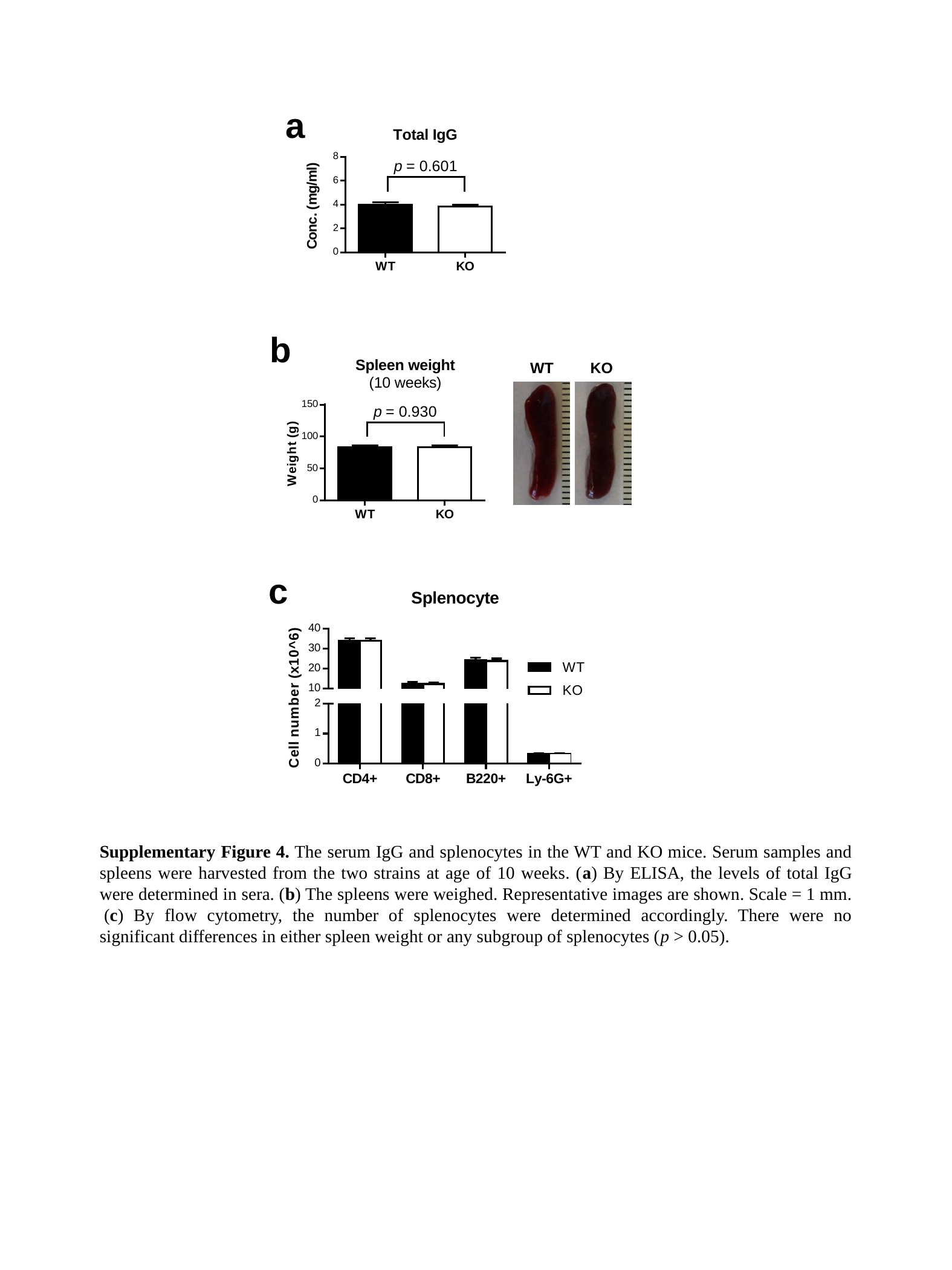

a
b
WT
KO
c
Supplementary Figure 4. The serum IgG and splenocytes in the WT and KO mice. Serum samples and spleens were harvested from the two strains at age of 10 weeks. (a) By ELISA, the levels of total IgG were determined in sera. (b) The spleens were weighed. Representative images are shown. Scale = 1 mm.  (c) By flow cytometry, the number of splenocytes were determined accordingly. There were no significant differences in either spleen weight or any subgroup of splenocytes (p > 0.05).

## Slide 5
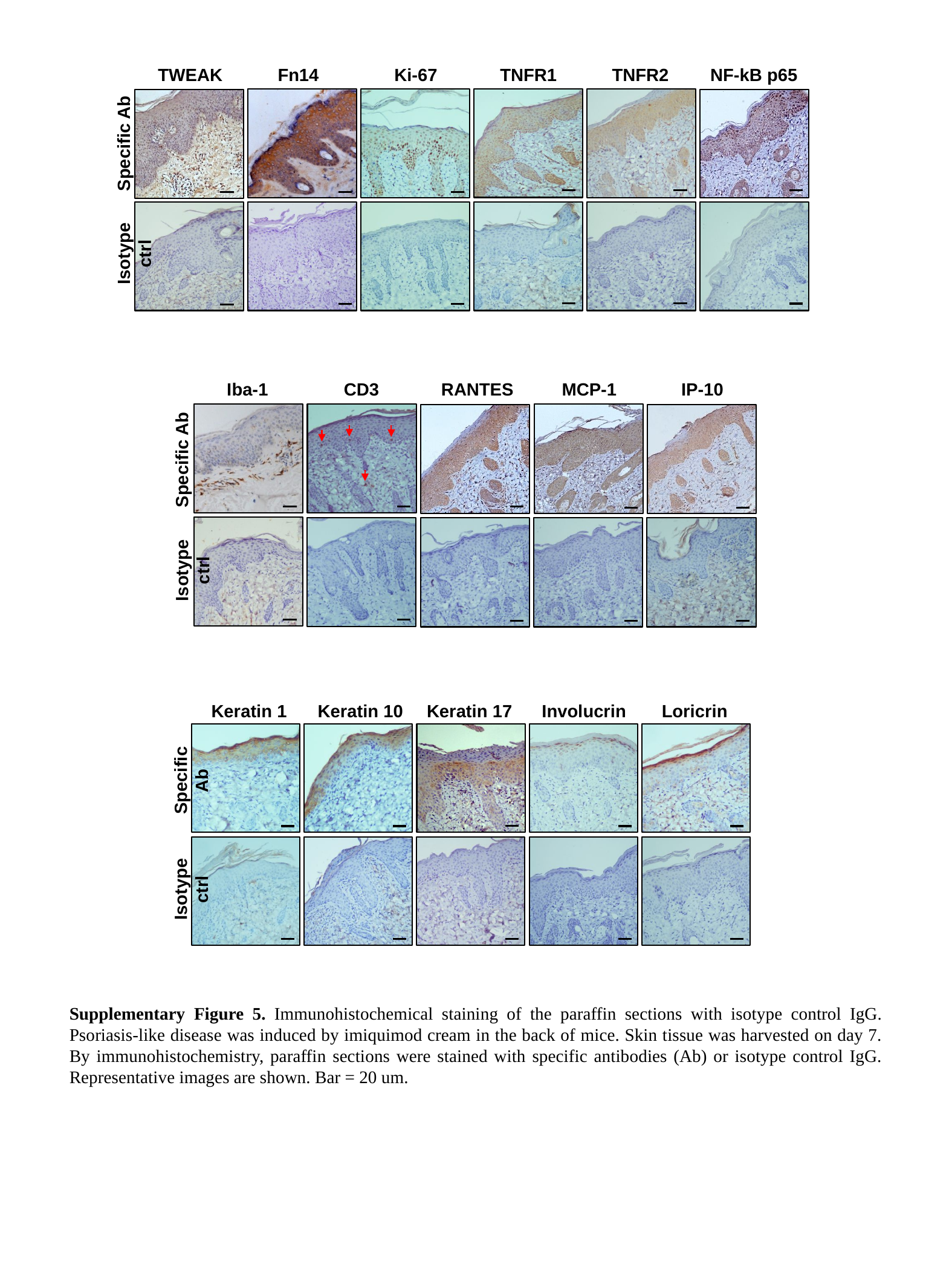

Fn14
Ki-67
TNFR1
TNFR2
NF-kB p65
TWEAK
Specific Ab
Isotype ctrl
Iba-1
RANTES
MCP-1
IP-10
CD3
Specific Ab
Isotype ctrl
Keratin 1
Keratin 10
Keratin 17
Involucrin
Loricrin
Specific Ab
Isotype ctrl
Supplementary Figure 5. Immunohistochemical staining of the paraffin sections with isotype control IgG. Psoriasis-like disease was induced by imiquimod cream in the back of mice. Skin tissue was harvested on day 7. By immunohistochemistry, paraffin sections were stained with specific antibodies (Ab) or isotype control IgG. Representative images are shown. Bar = 20 um.

## Slide 6
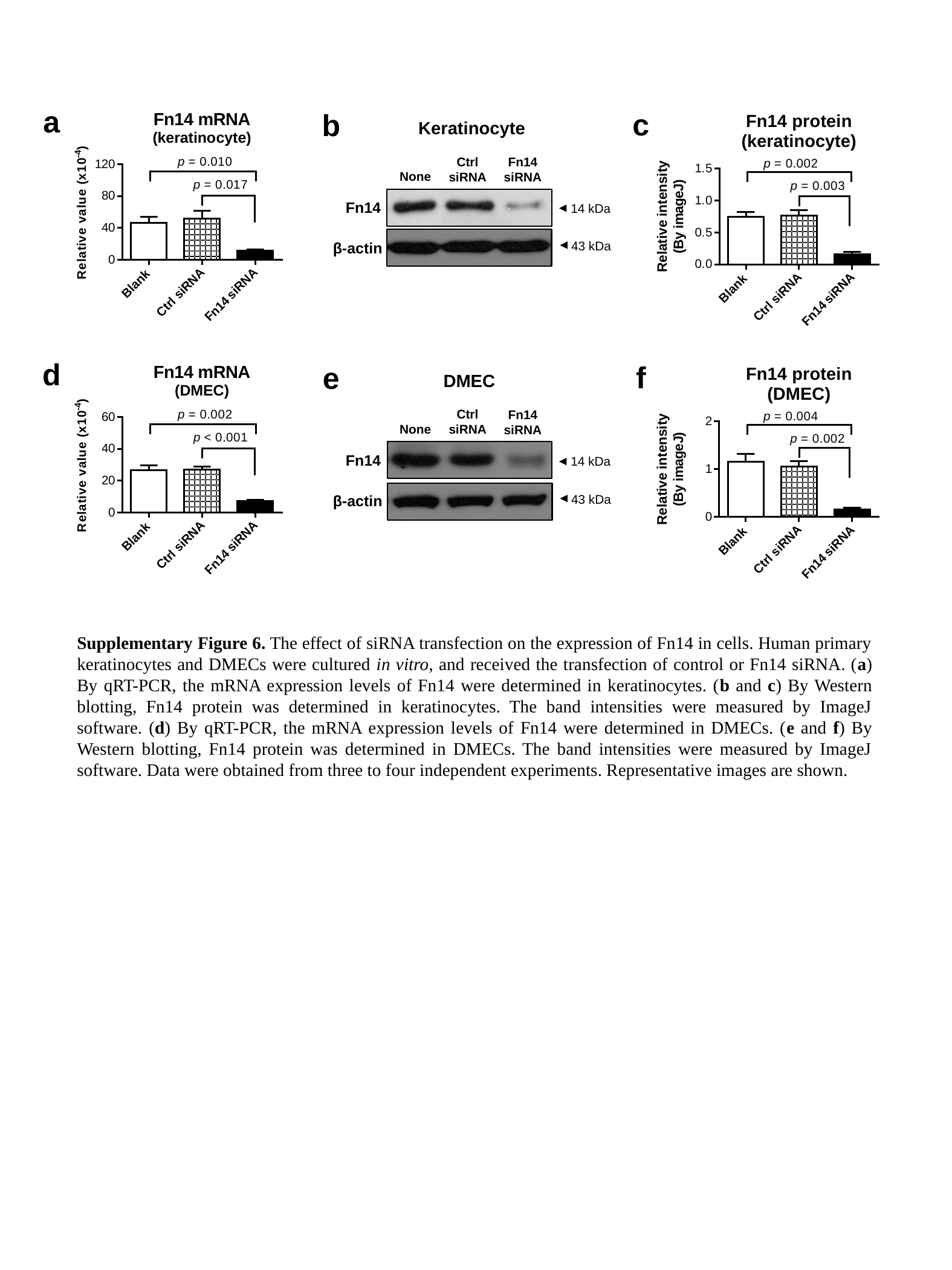

a
c
b
Keratinocyte
Ctrl
siRNA
Fn14
siRNA
None
Fn14
β-actin
14 kDa
43 kDa
d
f
e
DMEC
Ctrl
siRNA
Fn14
siRNA
None
Fn14
β-actin
14 kDa
43 kDa
Supplementary Figure 6. The effect of siRNA transfection on the expression of Fn14 in cells. Human primary keratinocytes and DMECs were cultured in vitro, and received the transfection of control or Fn14 siRNA. (a) By qRT-PCR, the mRNA expression levels of Fn14 were determined in keratinocytes. (b and c) By Western blotting, Fn14 protein was determined in keratinocytes. The band intensities were measured by ImageJ software. (d) By qRT-PCR, the mRNA expression levels of Fn14 were determined in DMECs. (e and f) By Western blotting, Fn14 protein was determined in DMECs. The band intensities were measured by ImageJ software. Data were obtained from three to four independent experiments. Representative images are shown.

## Slide 7
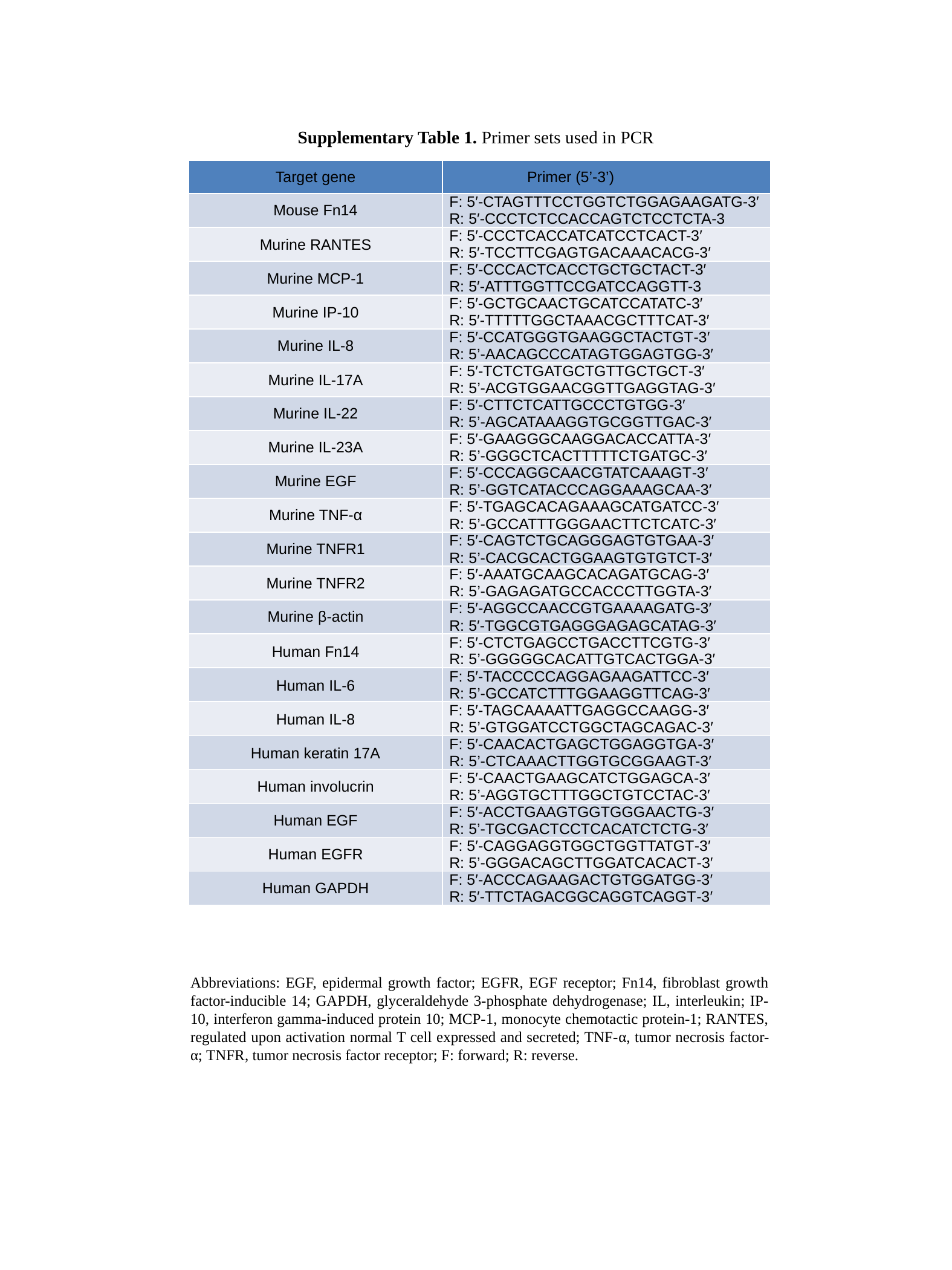

Supplementary Table 1. Primer sets used in PCR
| Target gene | Primer (5’-3’) |
| --- | --- |
| Mouse Fn14 | F: 5′-CTAGTTTCCTGGTCTGGAGAAGATG-3′ R: 5′-CCCTCTCCACCAGTCTCCTCTA-3 |
| Murine RANTES | F: 5′-CCCTCACCATCATCCTCACT-3′ R: 5′-TCCTTCGAGTGACAAACACG-3′ |
| Murine MCP-1 | F: 5′-CCCACTCACCTGCTGCTACT-3′ R: 5′-ATTTGGTTCCGATCCAGGTT-3 |
| Murine IP-10 | F: 5′-GCTGCAACTGCATCCATATC-3′ R: 5′-TTTTTGGCTAAACGCTTTCAT-3′ |
| Murine IL-8 | F: 5′-CCATGGGTGAAGGCTACTGT-3′ R: 5’-AACAGCCCATAGTGGAGTGG-3′ |
| Murine IL-17A | F: 5′-TCTCTGATGCTGTTGCTGCT-3′ R: 5’-ACGTGGAACGGTTGAGGTAG-3′ |
| Murine IL-22 | F: 5′-CTTCTCATTGCCCTGTGG-3′ R: 5’-AGCATAAAGGTGCGGTTGAC-3′ |
| Murine IL-23A | F: 5′-GAAGGGCAAGGACACCATTA-3′ R: 5’-GGGCTCACTTTTTCTGATGC-3′ |
| Murine EGF | F: 5′-CCCAGGCAACGTATCAAAGT-3′ R: 5’-GGTCATACCCAGGAAAGCAA-3′ |
| Murine TNF-α | F: 5′-TGAGCACAGAAAGCATGATCC-3′ R: 5’-GCCATTTGGGAACTTCTCATC-3′ |
| Murine TNFR1 | F: 5′-CAGTCTGCAGGGAGTGTGAA-3′ R: 5’-CACGCACTGGAAGTGTGTCT-3′ |
| Murine TNFR2 | F: 5′-AAATGCAAGCACAGATGCAG-3′ R: 5’-GAGAGATGCCACCCTTGGTA-3′ |
| Murine β-actin | F: 5′-AGGCCAACCGTGAAAAGATG-3′ R: 5′-TGGCGTGAGGGAGAGCATAG-3′ |
| Human Fn14 | F: 5′-CTCTGAGCCTGACCTTCGTG-3′ R: 5’-GGGGGCACATTGTCACTGGA-3′ |
| Human IL-6 | F: 5′-TACCCCCAGGAGAAGATTCC-3′ R: 5’-GCCATCTTTGGAAGGTTCAG-3′ |
| Human IL-8 | F: 5′-TAGCAAAATTGAGGCCAAGG-3′ R: 5’-GTGGATCCTGGCTAGCAGAC-3′ |
| Human keratin 17A | F: 5′-CAACACTGAGCTGGAGGTGA-3′ R: 5’-CTCAAACTTGGTGCGGAAGT-3′ |
| Human involucrin | F: 5′-CAACTGAAGCATCTGGAGCA-3′ R: 5’-AGGTGCTTTGGCTGTCCTAC-3′ |
| Human EGF | F: 5′-ACCTGAAGTGGTGGGAACTG-3′ R: 5’-TGCGACTCCTCACATCTCTG-3′ |
| Human EGFR | F: 5′-CAGGAGGTGGCTGGTTATGT-3′ R: 5’-GGGACAGCTTGGATCACACT-3′ |
| Human GAPDH | F: 5′-ACCCAGAAGACTGTGGATGG-3′ R: 5′-TTCTAGACGGCAGGTCAGGT-3′ |
Abbreviations: EGF, epidermal growth factor; EGFR, EGF receptor; Fn14, fibroblast growth factor-inducible 14; GAPDH, glyceraldehyde 3-phosphate dehydrogenase; IL, interleukin; IP-10, interferon gamma-induced protein 10; MCP-1, monocyte chemotactic protein-1; RANTES, regulated upon activation normal T cell expressed and secreted; TNF-α, tumor necrosis factor-α; TNFR, tumor necrosis factor receptor; F: forward; R: reverse.
